# Daily Treatment of Mice with Type 2 Diabetes with Adropin for Four Weeks Improves Glucolipid Profile, Reduces Hepatic Lipid Content and Restores Elevated Hepatic Enzymes in Serum

**DOI:** 10.3390/ijms23179807

**Published:** 2022-08-29

**Authors:** Marek Skrzypski, Paweł A. Kołodziejski, Ewa Pruszyńska-Oszmałek, Tatiana Wojciechowicz, Paulina Janicka, Małgorzata Krążek, Emilian Małek, Mathias Z. Strowski, Krzysztof W. Nowak

**Affiliations:** 1 Department of Animal Physiology, Biochemistry and Biostructure, Poznan University of Life Sciences, Wolynska 35, 60-637 Poznan, Poland; 2 Department of Preclinical Sciences and Infectious Diseases, Poznan University of Life Sciences, Wolynska 35, 60-637 Poznan, Poland; 3 Department of Hepatology and Gastroenterology, Charité-University Medicine Berlin, 13353 Berlin, Germany; 4 Medical Clinic III, 15236 Frankfurt (Oder), Germany

**Keywords:** adipocytes, adropin, pancreatic islets, metabolism, type 2 diabetes mellitus

## Abstract

Adropin is a peptide hormone encoded by Energy Homeostasis Associated gene. Adropin modulates energy homeostasis and metabolism of lipids and carbohydrates. There is growing evidence demonstrating that adropin enhances insulin sensitivity and lowers hyperlipidemia in obese mice. The aim of this study was to investigate the effects of daily administration of adropin for four weeks in mice with experimentally induced type 2 diabetes (T2D). Adropin improved glucose control without modulating insulin sensitivity. Adropin reduced body weight, size of adipocytes, blood levels of triacylglycerol and cholesterol in T2D mice. T2D mice treated with adropin had lower liver mass, reduced hepatic content of triacylglycerol and cholesterol. Furthermore, adropin attenuated elevated blood levels of hepatic enzymes (ALT, AST, GGT and ALP) in T2D mice. In T2D mice, adropin increased the circulating adiponectin level. Adropin had no effects on circulating insulin and glucagon levels and did not alter pancreatic islets morphology. These results suggest that adropin improves glucose control, lipid metabolism and liver functions in T2D. In conjunction with reduced lipid content in hepatocytes, these results render adropin as an interesting candidate in therapy of T2D.

## 1. Introduction

Adropin is a peptide hormone encoded by Energy Homeostasis Gene [1]. Adropin is mainly produced in the liver and brain; nevertheless, it is also detected in other peripheral tissues such as pancreas as well as in the cardiovascular system [1,2,3]. Several studies provided evidence that the biological effects of adropin are mediated through activation of GPR19 receptor [4,5,6]. Nevertheless, it needs to be pointed out that adropin is considered not only as a peptide hormone but also as a membrane protein interacting with the brain-specific Notch1 ligand NB3 [7]. Lessons from animal studies provide convincing evidence that adropin is involved in controlling glucose and lipid metabolism.

Obesity is a major risk factor in T2D. Derangements of glucose and lipid metabolism with hyperglycemia and hyperlipidemia are hallmarks of T2D [8]. Insulin resistance contributes to hyperglycemia in the majority of patients with T2D [9]. In particular, increased lipid content of the liver (non-alcoholic fatty liver disease) is associated with peripheral insulin resistance [10]. The high content of lipids in hepatocytes is critically important in the pathophysiology of non-alcoholic steatohepatitis. Inflammation and high fat content in hepatocytes in steatohepatitis play a crucial role in the peripheral insulin resistance [11]. Elevated serum liver enzymes and—less frequently—C-reactive protein are surrogate parameters of steatohepatitis [12,13]. Despite years of research, currently there is no approved medication to reduce hepatic fat content and to improve inflammation in the liver. Therefore, agents with the ability to improve hepatic insulin resistance by reducing hepatic fat content, inflammation and proinflammatory cytokines are urgently needed to treat T2D and obesity. Several studies demonstrated that in humans, circulating adropin level is inversely correlated with body mass index (BMI) [14,15,16]. Furthermore, it has been found that adropin deficiency in mice is accompanied by adiposity as well as impaired insulin sensitivity [17]. By contrast, it has been reported that mice with high fat diet-induced obesity treated with exogenous adropin have improved insulin sensitivity and glucose tolerance [18]. Moreover, Akcilar et al. reported that intraperitoneal administration of adropin for 10 days improves lipid metabolism, reduces HOMA—IR, fasting glucose level and protects from body weight gain in rats with experimentally induced T2D [19]. Furthermore, these authors reported that rats with T2D treated with adropin had lower levels of TNF-alpha mRNA expression in the liver and reduced levels of liver enzymes in the circulation [19]. These results suggest a therapeutic potential of adropin in the treatment of T2D. By contrast the effects of adropin on glucose and insulin tolerance tests as well as pancreatic islets have not been studied so far. In the present study, we evaluated the effects of prolonged treatment of T2D mice with daily injections of adropin for four weeks on glucose tolerance, insulin sensitivity, body weight, lipid metabolism, circulating adipokines, insulin and glucagon as well as pancreatic islet morphology and the liver metabolic parameters.

## 2. Results

### 2.1. Adropin Improves Glucose Tolerance in T2D Mice

**Figure 1:**
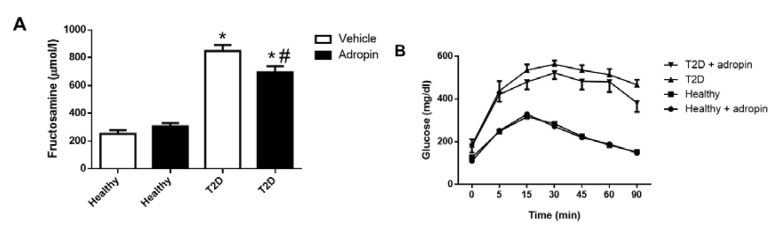

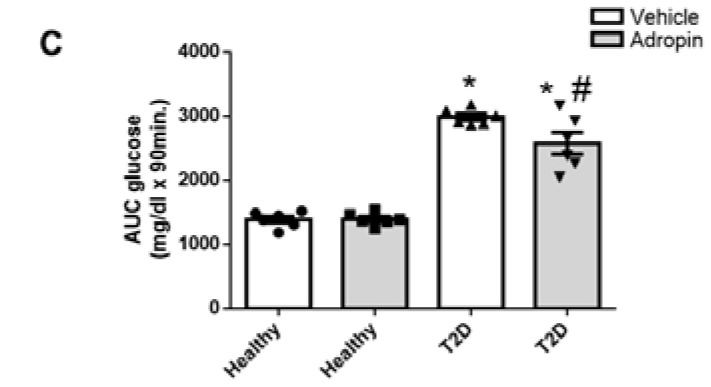
Effects of adropin on fructosamine level and glucose tolerance. (**A**) Fructosamine level in blood of healthy and T2D mice treated with vehicle or adropin (n = 12). (**B**) Glucose tolerance test performed in healthy and T2D mice treated with vehicle or adropin. (**C**) Calculated glucose AUC-90 in all experimental groups (n = 6). * *p* < 0.05 (vs. healthy PBS-treated mice). # *p* < 0.05 (vs. diabetic PBS-treated mice).

As shown in Figure 1A, induction of T2D in mice resulted in increased fructosamine levels as compared with control animals (847.8 ± 42.57 mg/dL vs. 253.2 ± 26.13 mg/dL, *p* ≤ 0.05). Adropin reduced fructosamine levels in diabetic animals (698.3 ± 42.35 mg/dL vs. 847.8 ± 42.57 mg/dL, *p* ≤ 0.05). In contrast, adropin had no effect on fructosamine levels in healthy mice. Diabetic mice treated with adropin had improved glucose tolerance as judged from decreased AUC for glucose (Figure 1B,C). These results indicate that adropin improves glucose control in T2D mice.

### 2.2. Adropin Treatment Fails to Improve Insulin Sensitivity in Healthy and Diabetic Mice

**Figure 2:**
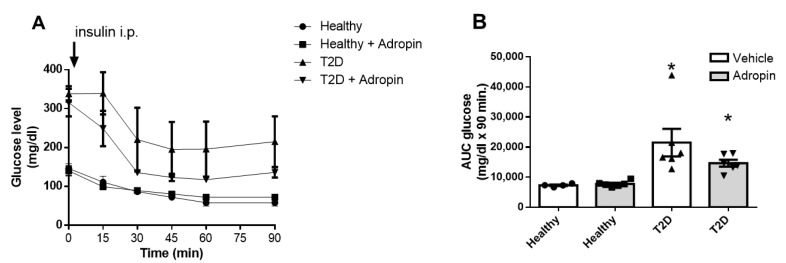
Effects of adropin on insulin tolerance in healthy and T2D mice. (**A**) Insulin tolerance test performed in healthy and T2D mice treated with vehicle or adropin. (**B**) Calculated glucose AUC-90 in all experimental groups (n = 4 (healthy PBS-treated mice) or n = 6). * *p* < 0.05 (vs. healthy PBS-treated mice).

Next, we performed insulin tolerance test in healthy and T2D mice treated with or without adropin. As shown in Figure 2A,B, mice with experimentally-induced T2D treated with or without adropin had reduced insulin sensitivity (*p* ≤ 0.05). Adropin failed to affect insulin sensitivity in both, healthy as well as T2D animals.

### 2.3. Adropin Does Not Modulate Insulin and Glucagon in Blood and Fails to Affect Alpha and Beta Cell Morphology in Healthy and T2D Mice

**Figure 3:**
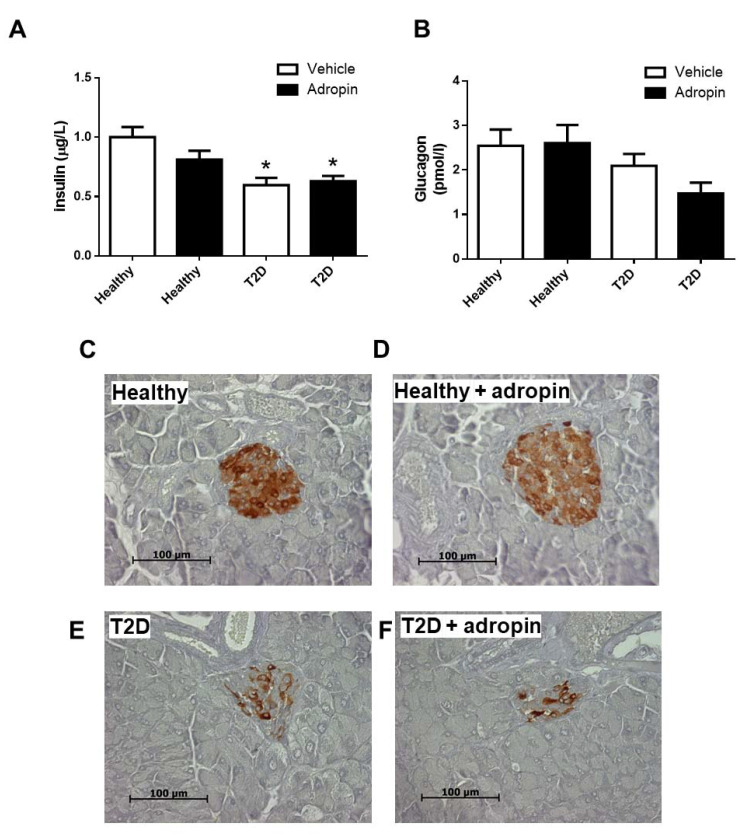

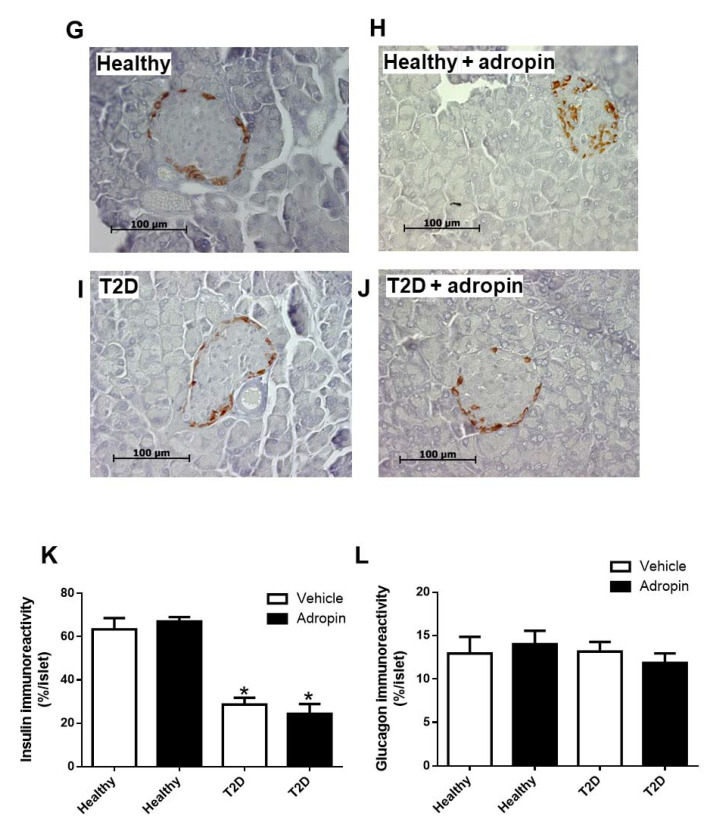
Effects of adropin on circulating insulin and glucagon levels as well as beta and alpha cell morphology. Plasma insulin (**A**) and glucagon (**B**) levels in healthy and T2D mice treated with vehicle or adropin (n = 12). Representative images of pancreatic islets stained with insulin antibody in healthy controls treated without (**C**) or with adropin (**D**) and in T2D mice treated without (**E**) or without adropin (**F**). Representative images of pancreatic islets stained with glucagon antibody in healthy controls treated without (**G**) or with adropin (**H**) and in T2D mice treated without (**I**) or without adropin (**J**). Quantification of insulin (**K**) and glucagon (**L**) immunoreactivity in healthy and T2D mice treated with or without adropin. * *p* < 0.05 (vs. healthy PBS-treated mice).

T2D mice had lower levels of insulin in the circulation as compared with healthy animals (Figure 3A). Circulating insulin levels were not affected by adropin, neither in healthy nor in diabetic animals (Figure 3A). By contrast, the levels of glucagon in the circulation were comparable in all experimental groups (Figure 3B).

Next, we assessed beta and alpha cell morphology in healthy and T2D mice. Insulin-immunoreactive area in pancreatic islets was reduced in mice with T2D (Figure 3E–K). However, adropin failed to affect beta cell morphology in both healthy and T2D mice (Figure 3C–K). In addition, pancreatic alpha cell morphology was not significantly different in all experimental groups (Figure 3G–L). These results indicate that exogenously administrated adropin does not modulate pancreatic alpha and beta cells in healthy or diabetic mice.

### 2.4. Adropin Attenuates Body Weight Gain and Improves Lipid Metabolism in T2D Mice

**Figure 4:**
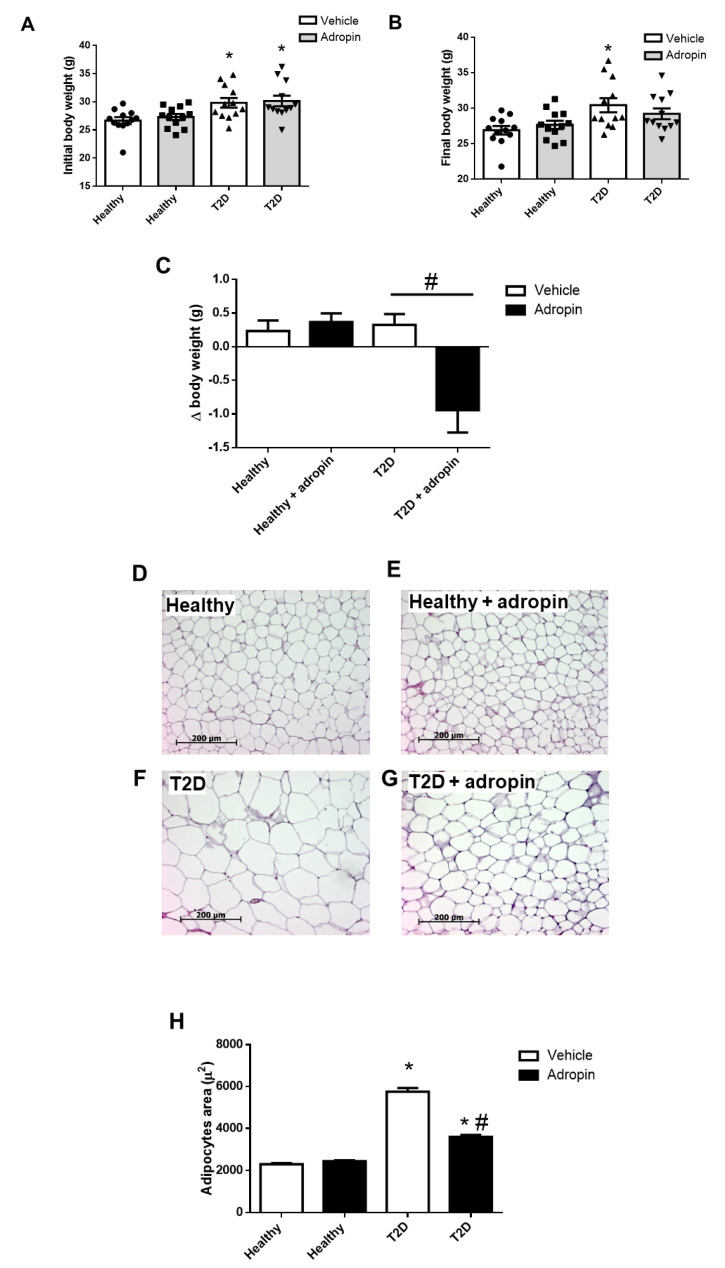

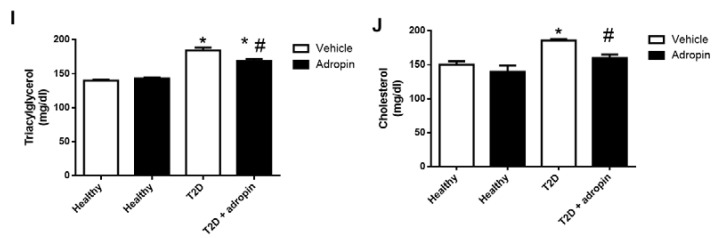
Effects of adropin on body weight, area of adipocytes and circulating triacylglycerol and cholesterol levels in healthy and T2D mice. (**A**) Initial body weight in all experimental groups before treatment with vehicle or adropin. (**B**) Final body weight in all experimental groups at the end of the experiment. (**C**) Δ body weight in all experimental groups (n = 12). Representative images of adipose tissue section from healthy animals treated without (**D**) or with adropin (**E**) and T2D mice treated without (**F**) or with adropin (**G**) (n = 200). (**H**) Size of adipocytes in all experimental groups. Serum levels of triacylglycerol (**I**) and cholesterol (**J**) in all experimental groups (n = 12). * *p* < 0.05 (vs. healthy PBS-treated mice). # *p* < 0.05 (vs. diabetic PBS-treated mice).

As shown in Figure 4A–C, exogenous adropin decreased body weight changes (Δ body weight), as compared to initial body weights in T2D. By contrast, adropin had no effect on body weight in healthy animals.

In addition, we studied adipocytes area in healthy and T2D mice. T2D mice (Figure 4F) had increased adipocytes as compared to healthy mice (Figure 4D). Adropin decreased the area of adipocytes in T2D mice (Figure 4G), whereas it had no effect on cell area in healthy mice (Figure 4E).

In addition, for the T2D mice treated with adropin, the elevated levels of triacylglycerol (Figure 4I) and cholesterol (Figure 4J) in the circulation were reduced.

### 2.5. Adropin Improves Liver Functions in T2D Mince

**Figure 5:**
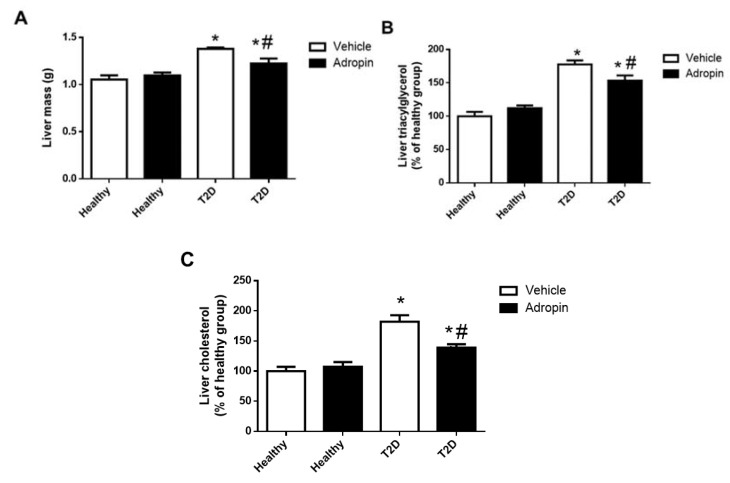

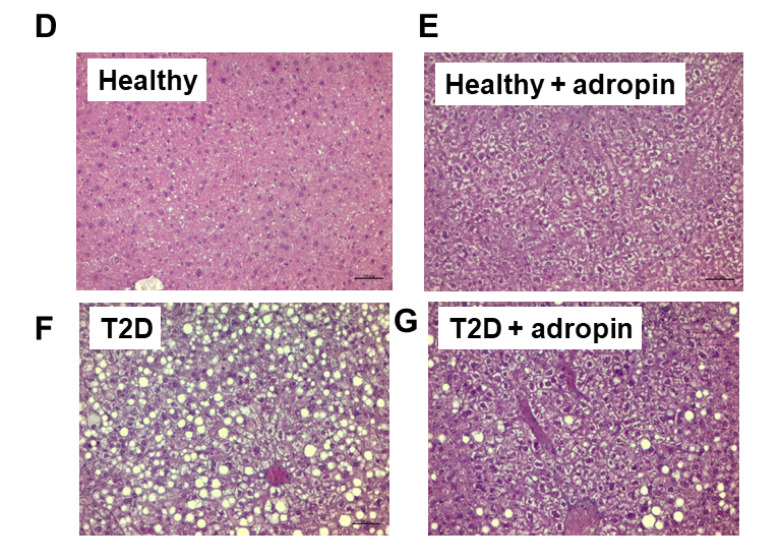
Effects of adropin on liver mass, hepatic content of triacylglycerol and cholesterol. (**A**) Liver mas in healthy and T2D mice treated with or without adropin. (**B**) Liver content of triacylglycerol in healthy and T2D mice treated with or without adropin. (**C**) Liver content of cholesterol in healthy and T2D mice treated with or without adropin (n = 10–12). (**D**–**G**) representative images showing HE staining of liver sections staining section in healthy animals treated without (**D**) or with adropin (**E**) and in T2D mice treated without (**F**) or with adropin (**G**). Intracellular vacuoles correspond to the levels of lipid accumulation. Magnification: 200× fold. * *p* < 0.05 (vs. healthy PBS-treated mice). # *p* < 0.05 (vs. diabetic PBS-treated mice).

T2D mice had increased liver mass, hepatic triacylglycerol and cholesterol content, as compared to healthy animals (Figure 5A–C). Adropin reduced liver mass, hepatic triacylglycerol and cholesterol content in diabetic mice (Figure 5A–C). Furthermore, we performed HE staining of fixed livers (Figure 5D–G). The amount of intracellular vacuoles corresponding to the levels of lipid accumulation was increased in T2D mice, however it was reduced in T2D mice treated with adropin. As shown in Table 1, elevated levels of hepatic enzymes (ALT, AST, GGT and ALP) in the circulation of T2D mice were reduced by adropin treatment.

### 2.6. Adropin Modulates Circulating Adiponectin But Not Leptin in T2D Mice

**Figure 6:**
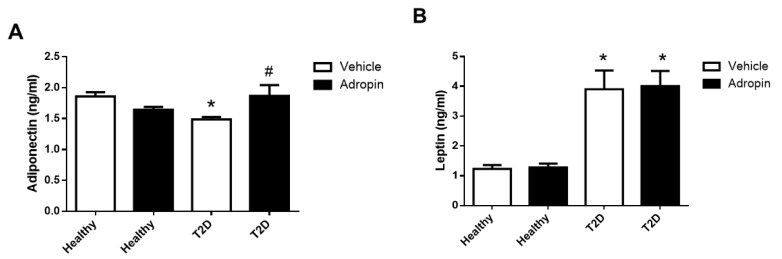
Effects of adropin on adiponectin and leptin in serum. Adiponectin (**A**) and leptin (**B**) concentration in healthy and T2D mice treated with or without adropin (n = 12). * *p* < 0.05 (vs. healthy PBS-treated mice). # *p* < 0.05 (vs. diabetic PBS-treated mice).

T2D mice had lower levels of adiponectin and higher levels of leptin in blood, as compared to healthy animals (Figure 6A,B). Adropin increased adiponectin level in the circulation of T2D mice (Figure 6A), but had no effect on leptin levels (Figure 6B).

## 3. Discussion

In the present study, we characterize the effects of chronic adropin administration in mice with experimentally induced T2D.

T2D is characterized by impaired glucose control and enhanced insulin resistance [9]. Here, we demonstrate that mice with experimentally induced T2D treated with adropin have decreased fructosamine levels (a surrogate marker for glycemic control [20]) and improved glucose tolerance. In the insulin tolerance test, there was a trend at increasing impaired insulin sensitivity in adropin-treated T2D mice; however, this effect was not statistically significant. It is worth noting that a previous study reported that adropin treatment for three days improved glucose control without affecting whole-body insulin sensitivity in mice fed a high-fat diet [21]. Importantly, the same study demonstrated that adropin effectively suppresses glucose production and enhances insulin sensitivity in the liver [21]. Therefore, it cannot be excluded that some of the beneficial effects of adropin observed in T2D mice resulted from improved liver functions.

In addition to impaired glucose control and insulin resistance, pancreatic beta cell loss and impaired insulin, and glucagon production secreted hallmark T2D [22,23]. As expected, we detected lower levels of insulin in the circulation in T2D animals. However, adropin treatment had no effects on insulin levels in diabetic animals. Furthermore, it is important to note that insulin in the circulation was lower in healthy animals treated with adropin; nevertheless, the difference was not statistically significant. In this context, it is important to note that in our previous in vitro study, we found that adropin suppresses insulin secretion from insulin producing INS-1E cells and from rat pancreatic islets [24]. Therefore, it is possible that adropin may also influence insulin secretion in vivo. Nevertheless, more studies are needed to verify this speculation. 

In contrast to insulin, circulating glucagon level was similar in all experimental groups. By contrast there are studies reporting elevated glucagon level glucagon in T2D patients [25]. Others reported that glucagon levels are not different in T2D individuals [26]. It cannot be excluded that normal glucagon levels in our experimental model of T2D may be due to a relatively short duration of our experiment. 

Taken together, these results collectively suggest that the beneficial effects of adropin on glucose control are rather independent on insulin and glucagon regulation. 

To confirm this assumption, we next assessed the effects of adropin on morphology of pancreatic beta and alpha cells. Adropin failed to affect morphology of pancreatic beta and alpha cells which support the hypothesis that an improved glucose control in response to chronic adropin appears to be independent upon the pancreatic islet cells regulation. 

Obesity is a major risk factor for the development of T2D [27]. The loss of body weight in obese individuals delays the onset of diabetes and also improves glucose homeostasis in diabetic patients [28]. In our study, adropin-treated T2D mice exposed to high-fat diet lost weight, which was accompanied by decreased adipocyte area. Consistent with this observation, previous studies demonstrated that adropin is involved in the regulation of body weight. For example, it has been reported that decreased levels of adropin in the circulation increase the risk of weight gain in *Rhesus macaques* fed a high-sugar diet [29]. Moreover, attenuated body weight gain was also reported in adropin overexpressing mice fed a high-fat diet for three months [1]. Although we detected reduced adipocyte area in adropin-treated mice, the mechanism responsible for the anti-obesity effects of adropin in T2D mice remains largely unknown. Nevertheless, previous study reported that male mice overexpressing adropin display a modest increase in energy expenditure per gram of body weight or fat-free mass [1]. Furthermore, we reported earlier that, in vitro, adropin suppresses differentiation of white preadipocytes into mature adipocytes [30]. In addition, we showed that by acting on adipocytes adropin induces lipolysis and suppresses lipogenesis [31,32]. Therefore, it is possible that adropin may promote body weight loss by modulating energy expenditure and the morphology/differentiation or function of white adipose tissue. 

Alterations in body weight in T2D patients are often associated with dyslipidaemia, which is manifested by increased triacylglycerol and cholesterol level in the circulation as well as liver steatosis [33,34]. In our study, diabetic mice treated with adropin had lower levels of circulating triacylglycerol as well as total cholesterol. Furthermore, we observed that T2D mice treated with adropin had decreased liver mass as well as hepatic triacylglycerol and cholesterol content. Moreover, adropin-treated mice had reduced serum levels of hepatic enzymes (ALT, AST, GGT and ALP). Our data are consistent with these results, showing that in rats with hyperlipidemia, adropin reduces hepatic triacylglycerol and total cholesterol [19]. 

In addition, it was reported that adropin overexpression in mice is associated with reduced serum levels of triacylglycerol [1]. Discussing the effects of adropin on lipid metabolism it is important to note that exogenously administrated adropin attenuates expression of hepatic enzymes involved in de novo fatty acid synthesis [35]. Finally, we recently showed that adropin suppresses expression of genes involved in lipid synthesis and downregulates lipogenesis in white adipocytes [30]. These data suggest that adropin attenuates lipid abnormalities in T2D mice. 

Obesity and T2D are characterized by alterations in adipokines productions [36]. Both adiponectin and leptin are relevant in controlling glucose and lipid metabolism in T2D [37]. In our study, T2D mice had lower serum levels of adropin and higher levels of leptin, which is consistent with published observations in T2D patients [38,39]. We also observed that the reduction of adiponectin in the circulation was restored by adropin in T2D mice. By contrast, leptin levels were not affected by adropin treatment. Importantly, adiponectin is able to improve hepatic insulin sensitivity as well as suppress liver glucose production [40]. Therefore, it is possible that adropin-mediated adiponectin normalization may contribute to improved glucose tolerance observed in T2D mice. Nevertheless, more studies are needed to verify this speculation. 

Our study has several limitations. For example, we did not assess body composition in all experimental groups. Moreover, food intake and energy expenditure were not investigated in this study. Furthermore, rodent model of diabetes, which was used in our study, does not completely reflect the human condition [41]. Therefore, any conclusions supporting a role of adropin in the treatment of human T2D patients requires further studies. 

In summary, we demonstrate here that adropin improves glucose control without affecting systemic insulin sensitivity and pancreatic alpha and beta cell morphology. Furthermore, our results show that adropin improves lipid metabolism, liver functions and increases adiponectin in T2D mice. We cautiously speculate that these results indicate that adropin may be considered as an interesting player in therapy of T2D.

## 4. Materials and Methods

### 4.1. Materials

Adropin^34–76^ (>98%) was obtained from Novazym (Poznan, Poland). Unless otherwise specified, all other reagents were purchased from Sigma-Aldrich (Darmstadt, Germany).

### 4.2. Animals

C57BJ/6 male mice were obtained from the Mossakowski Medical Research Centre Polish Academy of Sciences (Warsaw, Poland). Animals were housed under standard conditions (12/12 h light/dark cycle, 21 ± 1 °C).

### 4.3. Induction of T2D

After 2 weeks of acclimatization, 8 week-old mice weighing 20 ± 2 g were divided into two groups. One group (n = 24) was fed normal diet and the second group (n = 24) was fed high-fat diet (HFD) providing 50% energy from fat (ZooLab, Sędziszów, Poland). Both groups were fed ad libitum for 10 weeks. After 10 weeks, HFD animals were injected with a single dose of streptozotocin (50 mg/kg body weight). After 3 days, non-fasting blood glucose levels were measured in all animals. If blood glucose level was lower than 10 mmol/L, the injection of STZ (50 mg/kg body weight) was repeated. Next, healthy and diabetic groups were randomly divided into two groups (n = 12 in each group). Healthy and diabetic animals were fed a normal or high fat diet for four weeks and treated with or without adropin for four weeks, as described below.

### 4.4. Adropin Treatment

Adropin (100 nmol/kg of body weight) was injected intraperitoneally (i.p.) daily for a total of four weeks. Control animals were treated with the same volume of phosphate buffered saline (PBS), as vehicle. Animals were fasted for three hours prior to sacrifice. Collected tissues were frozen in liquid nitrogen and stored at −80 °C. Blood plasma was aliquoted and stored at −80 °C.

### 4.5. Intraperitoneal Glucose Tolerance (ipGTT) and Insulin Tolerance (ipITT) Tests

ipGTT and ipITT were performed as previously described [42].

### 4.6. Determination of Metabolic and Hormonal Profiles in Serum

Metabolic profile was assessed using colorimetric assays according to the manufacturer’s protocol. The levels of triglycerides (TG; Cat. No.: T7531), total cholesterol (Cat. No.: C7510), and fructosamine (Cat. No.: F7546) were measured using assay kits purchased from Pointe Scientific (Warsaw, Poland). The activities of alanine aminotransferase (ALT; Cat. No.: A7526), aspartate aminotransferase (AST; Cat. No.: A7561), γ-glutamyltransferase (γ-GTP; Cat. No.: G7571) and alkaline phosphatase (ALP, Cat. No.: AU400) in the serum were determined using Pointe Scientific assays (Pointe Scientific). Glucagon (Cat. No.: 10-1281-01) and insulin ultrasensitive ELISA kits (Cat. No.: 10-1249-01) were from Mercodia (Uppsala, Sweden). Leptin was determined using Mouse Leptin ELISA Kit (Cat. No.: EMD Millipore Corporation, St. Louis, MO, USA).

### 4.7. Immunocytochemistry and Histology

In brief, pancreatic tissue fragments were fixed in Bouin solution, then embedded in paraffin and cut into 3–4 μm sections. After deparaffinization (56 °C, 45 min, xylene 30 min), the sections were rehydrated. Antigenic sites were unmasked by warming three times for 5 min in 0.01 mol/l citrate buffer (pH 6.0) in a microwave oven. After cooling at room temperature, the sections were washed in PBST (pH 7.4) and incubated for 15 min with 3% H_2_O_2_ (vol./vol.). Next, sections were incubated for one hour with primary antibodies diluted 1:400 (anti-insulin sc-7839, Santa Cruz Biotechnology (Dallas, TX, USA); anti-glucagon SAB4501137, Sigma-Aldrich) and for 30 min with a secondary antibody biotinylated complex, and then for 30 min with an enzyme label streptavidin-HRP (DAKO LSAB 2 System™ HRP, Dako North America, Carpinteria, CA, USA). Immunoreactivity was revealed using 3,3′-diaminobenzidine as the chromogen and counterstained with Hematoxylin Solution Gill No. 2 for 30 s, washed gently in distilled water, and 5 min in tap water. Next, sections were dehydrated in the increasing ethanol series and incubated for 10 min in xylene, finished by coverslip sealed mounting using Roti Histokit (Roth Industries GmbH & Co. KG, Frederikssund, Denmark). The specificity of immunohistochemical staining was tested by omitting primary antibodies. These tests showed no staining. Percent of insulin and glucagon immunoreactivity was evaluated using ImageJ 1.53k software (National Institutes of Health, Bethesda, MD, USA).

To evaluate the area of adipocytes, epididymal adipose tissue fragments were fixed Bouin’s solution, embedded in paraffin, cut into 3-μm-thick sections and stained with hematoxylin and eosin (HE). Adipocytes area was determined using an LSM 510 inverted microscopy and Axio Vision Rel. version 4.6 software (Carl Zeiss, Oberkochen, Germany). HE staining of liver was performed the same.

### 4.8. Determination of Hepatic Triglycerides and Cholesterol Content

Lipid extraction was performed using the Folch method [43]. In brief, liver fragments weighing about 50 mg from the same site were homogenized using a Tissuelyser II ball homogenizer (Qiagen, Hilden, Germany) in a Folch mixture in a volume of 1 mL. Next, homogenates were vortexed for about 20 min and centrifuged at 4 °C at 5000× *g*. Then the supernatant was transferred to the new tubes and mixed with 0.2 mL of H_2_O. Probes were centrifuged again, and the upper phase was aspirated. A total of 20 µL of lower phase were transferred into the new tubes. In the next stage, the samples were evaporated using a vacuum concentrator (Eppendorf, Hamburg, Germany). After evaporation into the tubes, 150 µL of cholesterol or triglyceride reagent were added and samples were mixed for 10 min. After this time, 100 µL aliquots from each tube were transferred to a 96-well plate, and the absorbance was read using a Synergy 2 Biotek microplate reader (Agilent Technologies, Inc., Santa Clara, CA, USA).

### 4.9. Statistical Analysis

Data are demonstrated as the mean ± standard error of the mean. ANOVA followed by the Bonferroni post hoc test for comparison between groups was used. *p* < 0.05 (*) was considered to indicate a statistically significant difference.

## Figures and Tables

**Table 1:** The levels of ALT, AST, GGT and ALP in serum of healthy and T2D mice treated with or without adropin (n = 12). * *p* < 0.05 (vs. healthy PBS-treated mice). # *p* < 0.05 (vs. diabetic PBS-treated mice).

Groups	Healthy			T2D
	Vehicle	Adropin	Vehicle	Adropin
Parameter (IU/L)				
ALT	16.75 ± 0.53	16.85 ± 0.67	28.73 ± 0.63 (*)	22.25 ± 1.44 (*,#)
AST	60.94 ± 1.64	66.83 ± 2.14	92.7 ± 1.95 (*)	82.68 ± 3.3 (*, #)
GGT	9.21 ± 1.26	8.29 ± 1.26	18.52 ± 1.94 (*)	12.68 ± 1.68 (#)
ALP	57.96 ± 5.62	62.97 ± 4.05	79.19 ± 3.18 (*)	62.51 ± 3.42 (#)

## Data Availability

The data are available on reasonable request from the corresponding author.

## Abbreviations

ALP	alkaline phosphatase
ALT	alanine transaminase
AST	aspartate transaminase
BMI	body mass index
GGT	gamma-glutamyltransferase
GPR19	G Protein-Coupled Receptor 19
HOMA-IR	homeostatic Model Assessment for Insulin Resistance
NB3	contactin 6
PBS	phosphate-buffered saline
T2D	type 2 diabetes
TNF-alpha	tumor necrosis factor alpha
